# Combination effect of Chinese kidney-tonifying granules and platelet-rich plasma gels on enhancing bone healing in rat models with femur defects

**DOI:** 10.1186/s13018-023-04468-z

**Published:** 2023-12-19

**Authors:** Zhiqian Huo, Feng Wu, Guoliang Lu, Feng Huang

**Affiliations:** 1 Major in Orthopaedics of Traditional Chinese Medicine, The First Clinical College, Guangzhou University of Chinese Medicine, Guangzhou, 510006 Guangdong Province China; 2 Sixth Department of Orthopedics & Traumatology, Foshan Hospital of Traditional Chinese Medicine Affiliated to Guangzhou University of Chinese Medicine, Foshan, 528000 Guangdong Province China; 3 Prestigious Chinese Medicine Expert of Guangdong Province Xu Zhiqiang Inheritance Studio, Foshan, 528000 Guangdong Province China; 4 Department of Orthopedics, The First Affiliated Hospital of Guangzhou University of Chinese Medicine, Guangzhou, 510405 Guangdong Province China

**Keywords:** Bone defects, Platelet-rich plasma, Bushen Zhongyao Keli, Runx2, Osterix; Tonifying kidneys

## Abstract

**Background:**

The traditional Chinese kidney-tonifying granules, known as Bushen Zhongyao Keli (BSZYKL), have been found to stimulate calcium salt deposition, enhance bone formation, and foster bone growth within the bone matrix at sites of bone defects. On the other hand, platelet-rich plasma (PRP) is enriched with various growth factors capable of facilitating the repair of bone defects and enhancing bone strength following fractures. This study is dedicated to investigating the combined efficacy of BSZYKL and PRP gel (PRP-G) in the treatment of bone defects.

**Methods:**

We established a femur defect model in male Sprague-Dawley (SD) rats and filled the defect areas with autologous coccygeal bone and PRP-G. For 8 consecutive weeks, those rats were given with intragastric administration of BSZYKL. Biomechanical characteristics of the femur were assessed 28 days after intramuscular administration. On day 56, bone formation was examined using X-ray, micro-CT, and transmission electron microscopy. Additionally, we analyzed the expression of bone formation markers, Runx2 and Osterix, in femur tissues through qPCR, Western blotting, and immunohistochemistry.

**Results:**

Rats receiving the combined treatment of BSZYKL and PRP-G exhibited drastically enhanced femoral peak torsion, failure angle, energy absorption capacity, and torsional stiffness as compared to control group. This combination therapy also led to marked improvements in bone volume, mass, and microarchitecture, accompanied by elevated expressions of Runx2 and Osterix when compared to control group. Notably, the synergistic effects of BSZYKL and PRP-G in treating bone defects surpassed the effects of either treatment alone.

**Conclusions:**

These findings revealed the potential of BSZYKL in combination with PRP-G in improving bone defects.

## Introduction

A bone defect represents a disruption of the structural integrity of bone. Its causes encompass congenital malformations, tumor resections, fractures, and infections [1]. When bone defects occur on articular surfaces, in proximity to joints, or at ligament junctions, they can give rise to a spectrum of issues demanding surgical intervention to facilitate healing. This presents a significant challenge to orthopedic surgeons [2]. At present, there are many treatment options for reconstruction of bone defects, but the most effective methods in China mainly include autogenous cancellous bone transplantation, vascularized bone transplantation, Ilizarov technique, and membrane induction technique. Autologous bone grafting is a commonly used technique for repairing bone defects, but it has limitations in terms of the amount of bone mass. Additionally, it may also easily cause donor-site diseases, including movement limitations, perceptual disturbances, and pains [3, 4]. Membrane induction technology has been widely used in clinical practice because of its operability, reliability and high efficiency [5]. However, its anti-infection effect is poor, and it is easy to cause infection in the bone defect site, resulting in local poor blood circulation, avascular necrosis and even sepsis. Systemic use of antibiotics cannot reach the lesion; the treatment effect is not good [6]. For local application of antibiotics, action time is short and easy to drug resistance, difficult to completely sterilize [7], so it is necessary to find an effective alternative method.

Traditional Chinese medicine (TCM) holds that the kidney governs bones and engenders marrow, which is hidden in the bone cavity to nourish the bone. This concept is known as “tonifying kidney and benefiting marrow”. The generation of marrow provides the material basis for the kidneys to govern the bones, and tonifying kidneys can strengthen tendons and bones. For example, Dai et al. have shown that Jianpi Bushen Formula (a kidney-nourishing and spleen-invigorating prescription) can substantially improve trabecular bone area percentage, trabecular thickness, and estrogen levels in ovariectomized (OVX) rats, demonstrating that tonifying kidneys can promote bone formation and prevent postmenopausal bone loss [8]. Likewise, Wang et al. have documented the notable impact of the Bushen Qiangdu Formula, a prescription known for its kidney-reinforcing and spine-strengthening properties, in effectively modulating bone metabolism. Their findings demonstrate its potential to ameliorate osteoporosis and mitigate bone loss in individuals with ankylosing spondylitis [9].

Research has substantiated that those growth factors released by platelets during the healing of fractures possess the remarkable ability to stimulate the proliferation and differentiation of mesenchymal cells and osteocytes, while also fostering tissue angiogenesis [10]. Concentration of growth factors is instrumental in expediting bone healing, while their absence would eventually result in bone nonunion [11]. Platelet-rich plasma (PRP) is platelet concentrate obtained through the centrifugation of whole blood, which contains a variety of high concentrations of growth factors [12]. The bioactive substances in PRP stimulate the division of various cells in the body, activate inflammatory cells, and jointly promote the healing of bone defects [13]^.^ After the resection of jaw cysts, application of PRP in the defect site resulted in notable enhancements in bone regeneration at 12 and 18 weeks post-surgery. This highlights PRP's capacity to stimulate early bone regeneration [14]. Furthermore, PRP may be synergistically employed with polymer materials to enhance the recovery of bone defects. A 3D printed polylactic acid (PLA) scaffold coated with PRP exhibits superior mechanical properties, cell adhesion, and mineralization, and supports bone regeneration more effectively than PLA scaffolds [15]. The combination of PRP lysate with gelatin and hydroxyphenylpropionic acid conjugate can more effectively promote vertical and horizontal bone regeneration in alveolar ridge defects [16].

Therefore, we speculated that the combination of the Bushen Zhongyao Keli (BSZYKL) (a kidney-tonifying prescription in granules) and PRP could serve as a therapy to promote the healing of bone defects, which might be superior to the efficacy of BSZYKL or PRP alone. Our study aims to investigate the intriguing effects of BSZYKL combined with PRP gel (PRP-G) on bone healing in rats with femur defects, with the goal of examining our hypothesis. We conducted a comprehensive array of cutting-edge techniques, including biological tensile tests, X-rays, micro-computed tomography (Micro-CT), transmission electron microscope scanning, quantitative PCR (qPCR), Western blotting, and immunohistochemistry (IHC) assays, to unravel the secrets of accelerated bone regeneration.

## Methods

### PRP-G preparation

Initially, 3 mL blood sample was collected from the orbital venous plexus of the rats and then centrifuged at 2300 × g for 10 s. Three layers from the bottom to the top were observed: a red blood cell layer, a buffycoat coat layer, and a platelet-poor plasma layer. Secondly, 1/3 of the lower platelet-poor plasma layer and a small part of the buffycoat layer were collected and centrifuged at 2200 × g/per mL for 2 min. The supernatant was discarded to for PRP collection. According to the volume ratio of thrombin to PRP = 1:10, PRP-G was formed after adding thrombin for 20–30 s.

### Bone defects model establishment

Thirty-two (the sample size is calculated based on T test, and the calculation formula is as follows: *n* = (*Z*_*α*_ + *Z*_*β*_)^2×^2*σ*^2/^*δ*^2^, n represents the number of samples in each group, Z_α_ and Z_β_ need to look up the table, σ represents the standard deviation, and δ represents the difference between the mean value of the treatment group and the control group) specific pathogen-free (SPF) male SD rats were divided into control, BSZYKL, PRP-G, and BSZYKL + PPR-G groups (*n* = 8). All the rats were abstained from food and drink for 12 h before surgery and were anesthetized by intraperitoneal injection of 3% pentobarbital sodium (1.5 mL/kg). The third distal coccygeal bone of the rat was removed in each group to prepare autologous bone grains, which were immersed in sterile normal saline for use. Subsequently, the right lower limb of each rat was disinfected with 75% ethanol. The skin and superficial fascia were cut sequentially along the lateral side of the right thigh, the tensor fasciae latae was bluntly separated, and the vastus lateralis and biceps femoris muscles were pulled laterally to expose the femur. A fretsaw was used to cut off the long bone defects area of about 10 mm in the middle of the femoral shaft. The surgical area was rinsed with normal saline, and the autologous bone grain was implanted and fixed in the bone defects area. Except for the control and BSZYKL groups, 0.3 mL PRP-G was implanted in the bone defects area and wrapped until it was completely covered, and 4–0 absorbable sutures (Vicryl, Johnson, USA) were used to suture the deep fascia and superficial fascia sequentially, and 1–0 silk sutures were used to suture the skin. Each rat was intramuscularly injected with penicillin 5 × 10^4^ U/d for 3 d after the operation to prevent infection.

### Preparation and administration of BSZYKL decoction

BSZYKL was supplied by Foshan Hospital of Traditional Chinese Medicine, Guangdong province, China. Its ingredients include Radix *Angelicae sinensis* 15 g, Radix *Angelicae pubescentis* 10 g, Radix *Achyranthis bidentatae* 10 g, Radix *Dipsaci* 15 g, Fructus *psoraleae* 15 g, Rhizoma *drynariae* 15 g, Rhizoma *cibotii* 15 g, and Cortex *eucommiae* 15 g. To prepare the BSZYKL decoction, 250 mL of water was added to the herbs and brought to a boil over high heat. Subsequently, the mixture was simmered for approximately 45 min under low heat. The resulting decoction was further concentrated to a final volume of 90 mL. The BSZYKL and BSZYKL + PRP-G groups were given BSZYKL decoction (10 mL/kg) intragastrically and a normal diet the next day after surgery, while the control and PRP-G groups were given an equal volume of normal saline. The administration lasted 8 weeks.

### Biomechanical test

On the 28th day of intragastric administration, two rats were randomly selected from each group for the biomechanical test. Additionally, two healthy rats (without bone defects) were included as a reference group. The femurs were removed immediately after the rats’ sacrifice, the surrounding soft tissue and periosteum were carefully removed, the femoral head was resected, and both ends of the femur were embedded in polymethylmethacrylate material. After the embedding was firm, the specimen was mounted on a multi-axial biomechanical testing machine, and the distal femur was rotated externally at l°/s until fracture. The peak torsion (Nm), failure angle (radians), energy to failure (Nm radian), and torsional stiffness (Nm/deg) were measured.

### X-ray and micro-CT

After 8 weeks of intragastric administration, the femurs of rats were taken and callus formation of rats in each group was detected by X-ray. Micro-CT was used to detect the bone microstructure indexes TV, BV, and TV/BV to assess bone reconstruction.

### Transmission electron microscope

After 8 weeks of intragastric administration, small pieces of the external callus tissues were taken from the rats, prepared into blocks at the size of 1.0–2.0 mm^3^, and placed in a decalcifying solution. The decalcifying solution was replaced twice a week until the specimen could be pierced with a needle. After rinsing, ethanol gradient dehydration, immersion, embedding, section, and staining, the ultrastructure of callus tissue cells was observed under a transmission electron microscope (SEM-JEOL5600, Japan).

### IHC

Femur tissue sections were blocked with 3% H_2_O_2_ for 1 h. After completely rinsing with 1 mol/L phosphate buffer (PBS), non-specific blocking serum was added and blocked at room temperature for 1 h. Then the primary antibody was added and incubated overnight at 4°C. The next day, after 3 times of washing with PBS, HRP Goat Anti-Rabbit IgG was added (A0208, Beyotime, China) and incubated at room temperature for 1 h. DAB was used for color development and the reaction was terminated by distilled water and then redyed with hematoxylin. Finally, the sections were observed, and graphs were captured with an inverted fluorescence microscope. Primary antibodies of Runx2 Rabbit pAb (A2851) and Anti-Sp7/Osterix antibody (Ab209484) were bought from Abclonal, China and Abcam, USA, respectively.

### qPCR

After 8 weeks of intragastric administration, the femur tissues of rats were milled with liquid nitrogen and total RNA was extracted by Trizol. RT reaction system (20 μL) was prepared on ice: Total RNA 2 μL, 5 × PrimeScriptTM Buffer 6 μL, Oligo dT Primer 1.5 μL, PrimeScriptTM RT Enzyme Mix I 1.5 μL, and DEPC 9 μL. The reverse transcription reaction was performed on a 9600 DNA amplification instrument (PE, USA). The qPCR reaction followed the procedure of the SYBR Premix Ex TaqTM PCR Kit (RR820A, Takara, Japan). The relative expression of each target gene was calculated using the 2^−∆∆Ct^ method. Primers sequences were: Runx2-F, CAAGGAGGCCCTGGTGTTTA; Runx2-R, CTCCAGAGCACTCACTGACTCG; Osterix-F, CAGCTGCCTACTTACCCGTC; Osterix-R, TCCAGTTGCCCACTATTGCC; GAPDH-F, CTTTGGTATCGTGGAAGGACTC, GAPDH-R, GTAGAGGCAGGGATGATGTTCT.

### Western blotting

After 8 weeks of intragastric administration, the total protein of the femur tissues was extracted and quantified. The protein sample was mixed with the loading buffer and denatured in a boiling water bath. The denatured protein samples were separated by gel electrophoresis and blocked with 5% skim milk powder after membrane transference. Primary antibody was added to the membrane for incubation at 4℃ overnight. HRP Goat Anti-Rabbit IgG (AS014, Abclonal, China) was added after washing with PBS and incubated at room temperature for 1 h. ECL luminescent solution (24,580, Thermo Fisher, USA) was used for luminescence, and then the protein bands were exposed to Gel Imager System. Primary antibodies Runx2 (A2851), Osterix (A18699), and GAPDH (A19056) were all purchased from Abclonal, China.

#### Statistical analysis

All data were processed through GraphPad Prism 9.0, and statistical results were expressed as mean value ± standard deviation. One-way ANOVA was used to analyze the data for homogeneity of variance. Data that did not satisfy homogeneity of variance were analyzed by Brown–Forsythe and Welch ANOVA tests. *P* value < 0.05 was considered as a significant difference between the two groups.

## Results

### Effect of BSZYKL combined with PRP-G on biomechanics characteristics

**Fig. 1:**
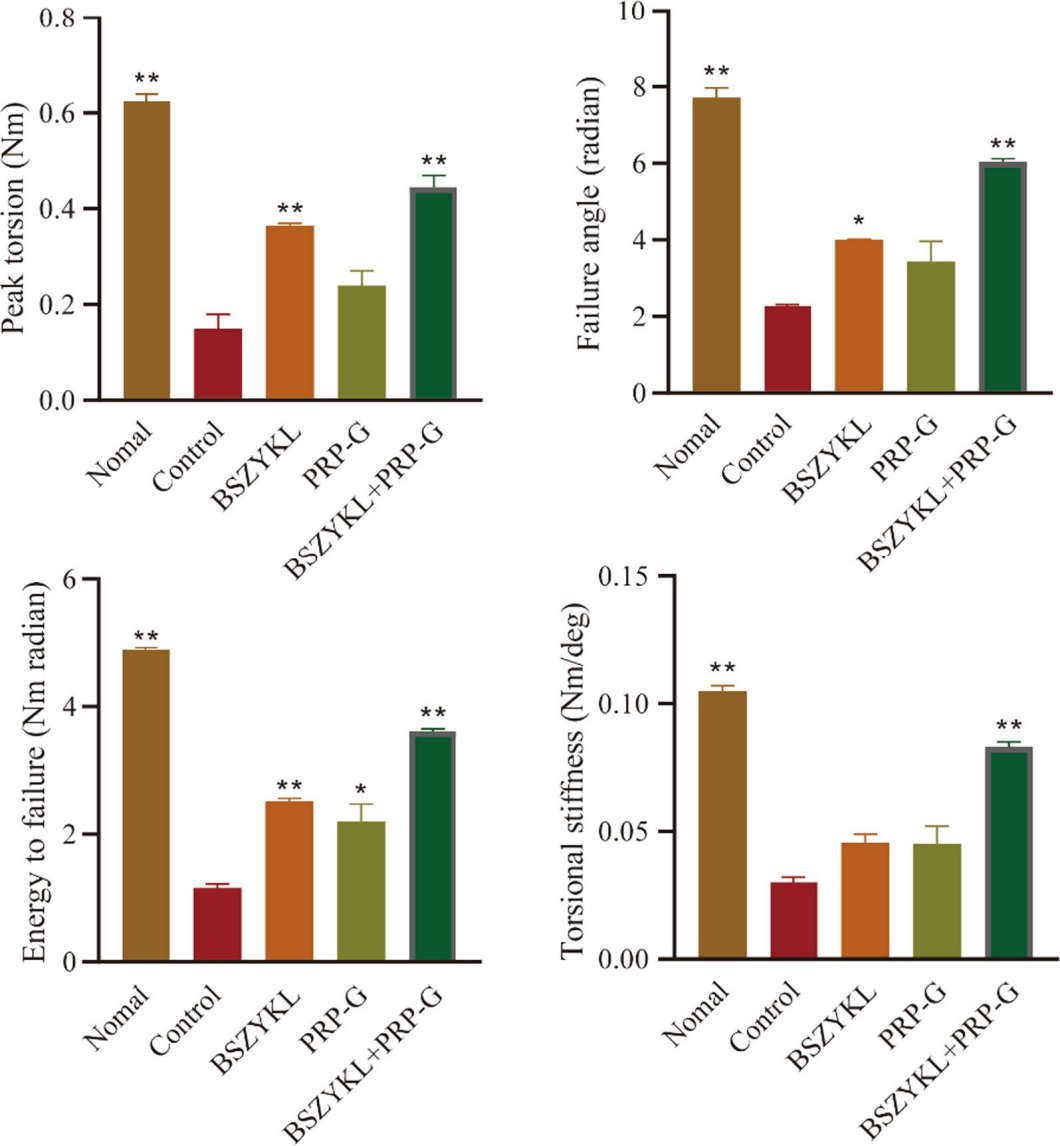
The biomechanics characteristics of peak torsion, failure angle, energy to failure, and torsional stiffness of the femur were measured on day 28. The combination of BSZYKL and PRP-G significantly enhanced biomechanics characteristics compared to the control group. ^*^P < 0.05, ^**^P < 0.01 *vs*. control group. Note: The control group refers to the model control group, which received the same amount of normal saline after establishing the rat bone defect model as the treatment group

Biomechanics characteristics are important indicators of bone growth and metabolism and are a comprehensive reflection of bone mass, bone structural continuity, bone cortical thickness, and bone material property. Bone tissues subjected to excessive stress may deform, and fractures may result when the stress exceeds a certain threshold [17]. The bone material property varies little with age and gender. The greater the bone mass, the greater the bone strength. Bone strength is an intrinsic characteristic of bone, independent of its size and shape [18]. On day 28, the rat femur was collected and biomechanics tests were performed to assess the bone strength in each group. Our findings revealed notable enhancements in femur strength among BSZYKL and BSZYKL + PRP-G groups, including increased peak torsion, failure angle, and energy to failure, all of which significantly surpassed those in control group. Furthermore, the energy to failure in RPR-G group and the torsional stiffness in BSZYKL + PRP-G group exhibited notable improvements compared to control group (Fig. 1).

### Effect of BSZYKL combined with PRP-G on bone healing

**Fig. 2:**
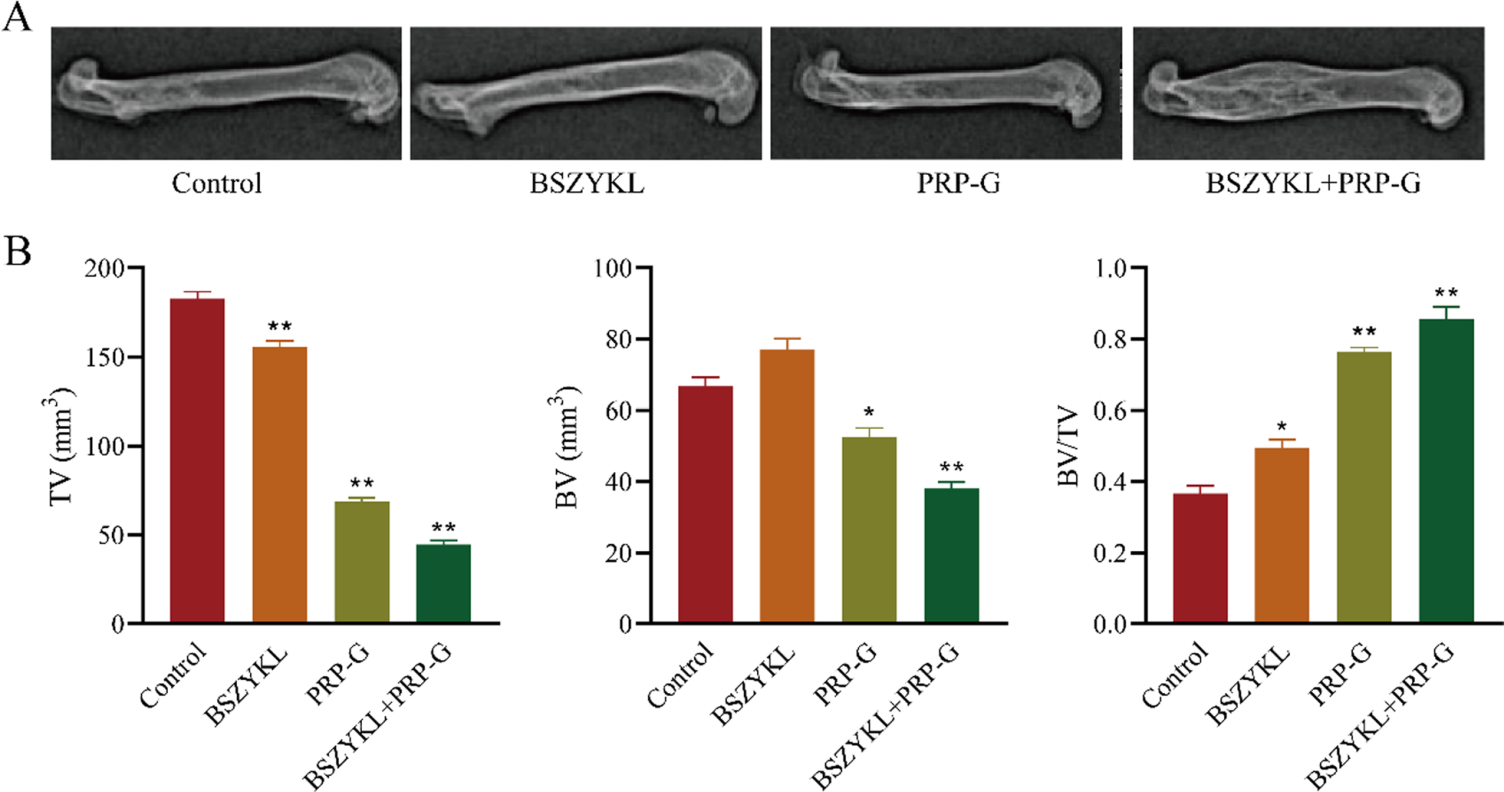
On day 56, the callus formation of the bone defects was observed by X-ray detection (**A**) and the bone microstructure indexes were measured by Micro-CT (**B**). ^*^P < 0.05, ^**^P < 0.01 *vs*. control group. Note: The control group refers to the model control group, which received the same amount of normal saline after establishing the rat bone defect model as the treatment group

Eight weeks after the administration, the femur of the rats was taken for X-ray examination. The results showed that in control group, the defect was clearly observed and a small amount of callus was formed. Callus formation showed superior results in both BSZYKL and PRP-G groups as compared to control group. A mass of callus formation was apparently observed in BSZYKL + PRP-G group (Fig. 2A). The micro-CT results indicated that the levels of TV in all three treatment groups decreased compared to control, while BV/TV levels increased (Fig. 2B). These results suggested that both BSZYKL and PRP-G promote bone healing and that the combination of BSZYKL and PRP-G is more effective than being applied alone.

### Effect of BSZYKL combined with PRP-G on the microarchitecture of osteocytes

**Fig. 3:**
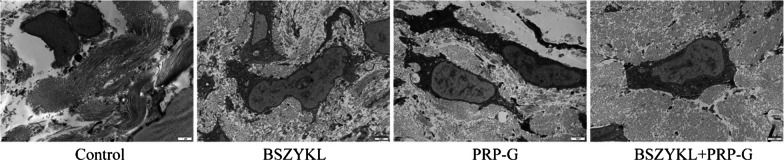
On day 56, the microarchitecture of the osteocytes was observed using a transmission electron microscope (12,000 × , 80 kV). Scale bar, 1 μm. Note: The control group refers to the model control group, which received the same amount of normal saline after establishing the rat bone defect model as the treatment group

In control group, large amounts of collagen fibers were absent around the osteocytes, and some of the collagen fibers were disordered with varying lengths. The cytoplasmic volume within the osteocytes was reduced. There were fewer cellular protuberances, and the internal organelles were few and inactive. In BSZYKL group, some collagen fibers were absent around the osteocytes. The nuclear membrane was concave, and there are fewer cellular protuberances. In PRP-G group, more collagen fibers were absent, and the nuclear membrane of osteocytes was slightly dented. Compared to control group, the cell protuberances increased, but free ribosomes decreased. In BSZYKL + PRP-G group, only a small amount of collagen fibers were absent around the osteocytes, and the collagen fibers were arranged densely and periodically. The shape of the nuclear membrane was normal. Compared with control group, there was an increase in cell protuberances and the number of organelles (Fig. 3).

### Effect of BSZYKL combined with PRP-G on expressions of Runx2 and Osterix

**Fig. 4:**
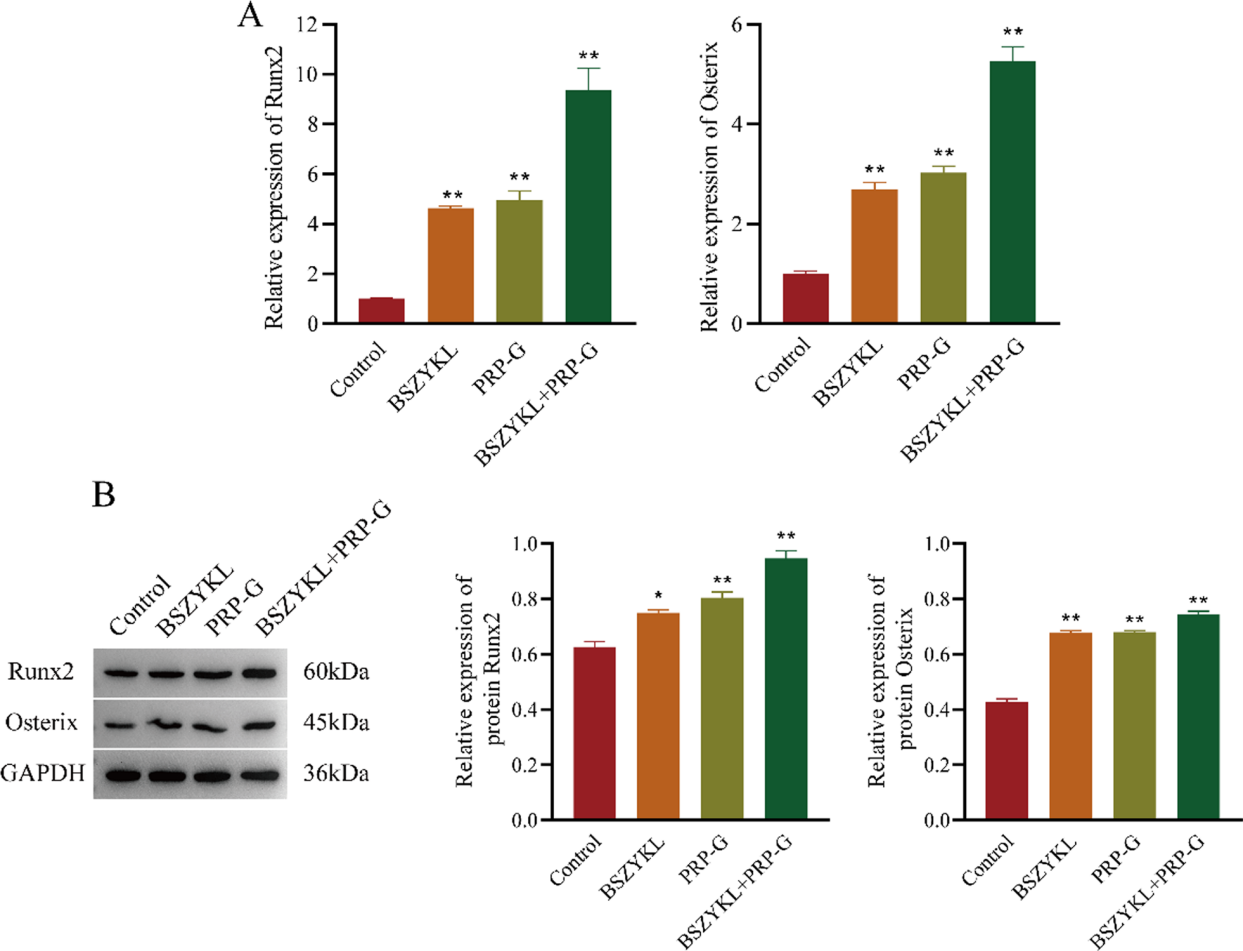
On day 56, the expression of Runx2 and Osterix in the bone tissue was determined by qPCR (**A**) and Western blotting (**B**). The combination of BSZYKL and PRP-G significantly increased the expression of Runx2 and Osterix compared to the control group. ^*^P < 0.05, ^**^P < 0.01 *vs*. control group. Note: The control group refers to the model control group, which received the same amount of normal saline after establishing the rat bone defect model as the treatment group

**Fig. 5:**
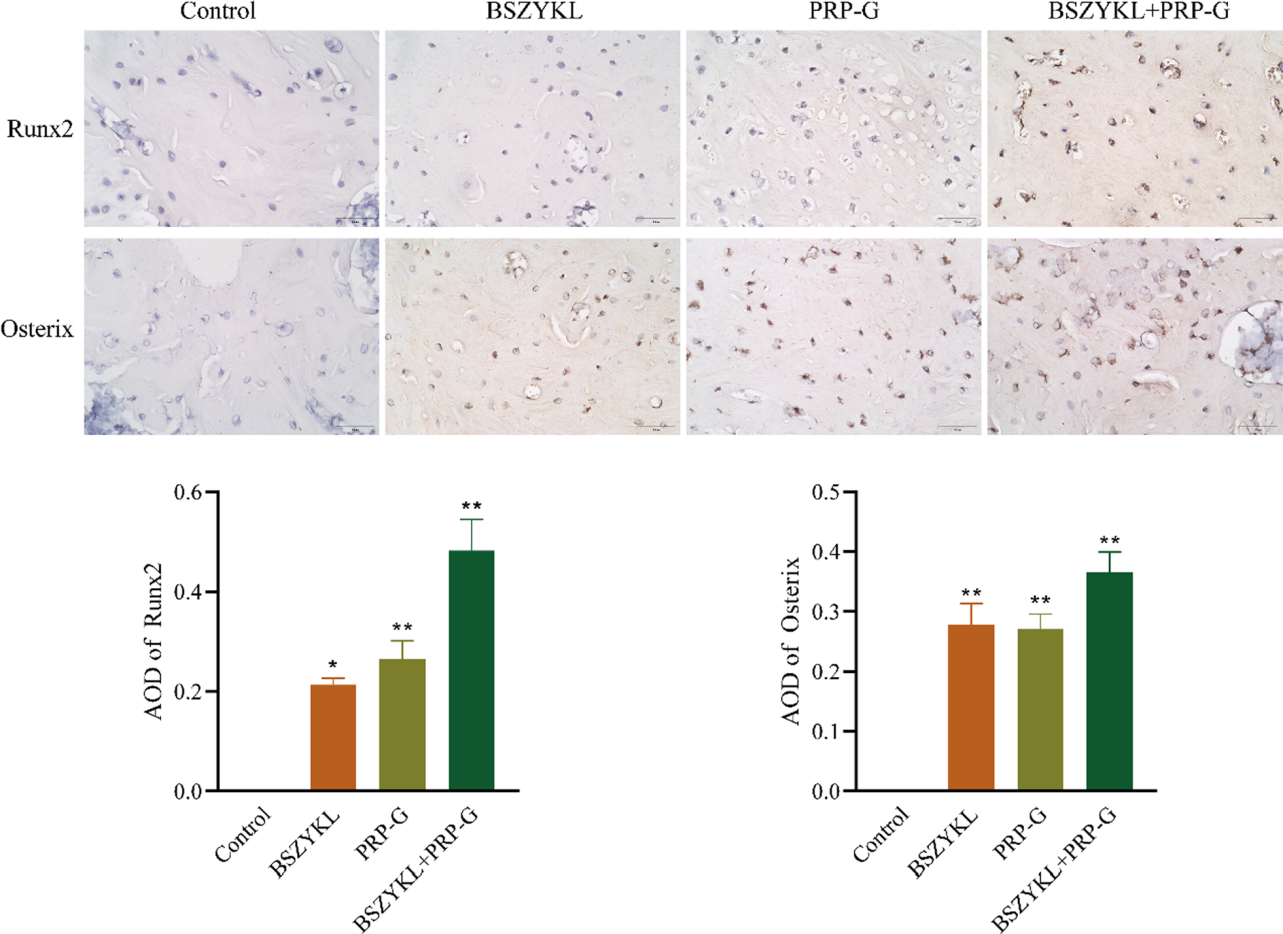
The expression and distribution of Runx2 and Osterix were determined by IHC. Compared to the control group, the combination of BSZYKL and PRP-G promoted the expression of Runx2 and Osterix in the bone tissue. Scale bar, 50 μm. * *P* < 0.05, ** *P* < 0.01 *vs*. control group. Note: The control group refers to the model control group, which received the same amount of normal saline after establishing the rat bone defect model as the treatment group

Runx2 serves as a marker for the initiation of osteoblast differentiation, while Osterix is a specific transcription factor that is essential for regulating osteoblast differentiation. After 8 weeks of administration, the expression of Runx2 and Osterix in the femur tissues of rats was detected by qPCR, Western blotting, and IHC. Results from qPCR and Western blotting showed significant increases in the expression of Runx2 and Osterix in all three treatment groups compared to control (Fig. 4A-B). Moreover, Runx2 and Osterix in BSZYKL + PRP-G group had the highest expression among these three groups. According to our IHC results, expression of the BSZYKL + PRP-G group was higher than the other three groups (Fig. 5). These results suggested that combination of BSZYKL and PRP-G promotes osteoblast differentiation by increasing the expression of Runx2 and Osterix, thus enhancing osteogenesis.

## Discussion

Concept of bone defects in TCM is not specifically described, but based on the symptoms, it belongs to the categories of “bone deficiency” and “bone atrophy”. Basic theory of TCM holds that the normal growth and development of bones are closely related to the functions of the kidney. Powerful kidney functions lead to robust marrow production and strong bones while kidney deficiency would ultimately result in bone fragility, fracture nonunion, and delayed healing. Kidney tonics are used to supplement kidney essence, nourish muscles, bones, and meridians, and promote the deposition of calcium salts in the bone matrix at the sites of bone defects, thereby enhancing bone formation and accelerating bone growth.

In this study, the monotherapy of PRP-G or BSZYKL promotes biomechanics characteristics, bone microstructure indexes, and callus formation (compared with control group). Nevertheless, the combined effect of BSZYKL and PRP-G outperforms that of BSZYKL and PRP-G alone. The BSZYKL is composed of Radix *A. sinensis*, Radix *A. pubescentis*, Radix *A. bidentatae*, Radix *Dipsaci*, Fructus *psoraleae*, Rhizoma *drynariae*, Rhizoma *cibotii*, and Cortex *eucommiae*. These herbs are effective in promoting bone formation and bone repair. Lin et. al found that water extract from Rhizoma *drynariae* increases bone mineral density in vertebrae and femur and greatly promotes osteogenesis in primary human osteoblasts, suggesting the osteogenic activity of Rhizoma *drynariae* [19]. Radix *Dipsaci*, the root of *Dipsacus asper* Wall. ex DC, has been shown to increase bone mineral content, bone density, and bone area in rats, and may be used in anti-osteoporosis therapy [20]. Cortex *eucommiae* markedly increases bone mineral density in the lumbar spine, femur, and tibia of OVX rats, suggesting that Cortex *eucommiae* could prevent postmenopausal osteoporosis [21]. Fructus *psoraleae* increases bone mass and promotes the differentiation of bone mesenchymal stem cells into osteoblasts in OVX rats [22]. Rhizoma *cibotii* strengthens bones and tendons and treats osteoporosis by activating the BMP2/SMAD1 signaling pathway for osteogenesis [23]. Radix *A. sinensis*, Radix *A. pubescentis*, and Radix *A. bidentatae* have also been proven to have an anti-osteoporosis effect and promote BMSC proliferation [24–26].

PRP contains a number of growth factors, such as platelet-derived growth factor, transforming growth factor β, vascular endothelial growth factor, epidermal growth factor, fibroblast growth factor, and insulin-like growth factor. These growth factors promote tissue repair and have been used extensively in the treatment of refractory wounds and the repair of oral and maxillofacial bone defects in recent years [27]. The research showed the usefulness of PRP in reducing subjective pain at the donor-site level after anterior cruciate ligament reconstruction with bone-patellar tendon-bone technique [28]. A study at the animal level showed that PRP promotes the healing of bone defects and improved bone strength after fracture healing [29]. The application of PRP to bone tissue engineering is wide and acceptable. For the repair of bone defects, activated PRP releases multiple growth factors through exocytosis, stimulating bone formation and accelerating bone repair [30]. Among the numerous growth factors released by PRP, platelet-derived growth factor BB plays a signaling role in regulating the secretion of osteoclast progenitor cells and promoting periosteum formation. It can also coordinate blood vessel formation and support bone growth during bone growth and remodeling [31]. Transforming growth factor *β* can promote the proliferation and differentiation of osteoblasts, regulate the synthesis of bone matrix, and coordinate bone resorption and bone formation [32]. The vascular endothelial growth factor promotes the migration, proliferation, and differentiation of vascular endothelial cells near the ossification center, stimulates the differentiation of osteoblasts and promotes the differentiation and survival of osteoclasts [33]. Fundamental fibroblast growth factor not only stimulates osteoclasts to enhance bone resorption but also promotes the proliferation and differentiation of osteoblasts, thus orchestrating a delicate balance in bone remodeling [34].

Additionally, the combination of BSZYKL and PRP-G significantly enhanced the expression of Runx2 and Osterix in the bone tissue of rats. Runx2 is a specific transcriptional regulator for mesenchymal stem cell differentiation into osteoblasts. It was found that Runx2-deficient mice (by gene knockout) did not undergo intramembranous or endochondral ossification due to their inability to differentiate effectively into osteoblasts, demonstrating Runx2 is an important player in the process of osteogenesis [35]. Runx2 regulates the expression of various specific markers of osteoblast proliferation and differentiation [36]. Runx2 expression is a marker for the initiation of osteoblast differentiation and induces the development of MSCs into osteoblasts or chondrocytes; thus, it is the earliest and most specific marker gene for bone formation [37]. Osterix is only expressed in developing bone tissue and it is an essential transcription factor for osteoblast differentiation and bone formation. It is also the most direct and specific transcription factor found to regulate osteoblast differentiation. The osteogenic ability of Osterix knockout mice was significantly lower than that of wild-type mice, which proved to play an important role in osteoblast maturation and terminal differentiation into osteoblasts [38].

In conclusion, our findings suggested that combination of BSZYKL and PRP-G may promote the proliferation and differentiation of osteoblasts by increasing the expression of Runx2 and Osterix, thus promoting bone formation. This study has found the potential of BSZYKL and PRP-G combined therapy in the treatment of bone defects, which provides an important theoretical and practical basis for developing new treatment schemes, optimizing clinical strategies and improving the treatment effect of bone defects.

However, this study also has some limitations. First, this study only investigated the effects of BSZYKL in male SD rat femur defect model. Although the animal model provides valuable information, it is necessary to verify these findings in human trials to ensure the translational relevance of these findings. In addition, although this study demonstrated that BSZYKL and PRP-G improved biomechanical properties, bone mineral density, and expression of markers of bone formation, the underlying molecular mechanisms by which BSZYKL and PRP-G exert their effects were not fully elucidated. Therefore, future research will focus on studying specific signaling pathways and molecular interactions. By addressing these limitations and conducting further studies, we can gain a deeper understanding of the efficacy, mechanisms and potential clinical applications of BSZYKL and PRP-G in combination for the treatment of bone defects.

## Data Availability

The data supporting the findings of the study are within the article.
